# Weight management practices, views, and experiences of adults living with poor socioeconomic circumstances and obesity: a qualitative systematic review protocol

**DOI:** 10.11124/JBIES-23-00238

**Published:** 2024-01-22

**Authors:** Jatta Salmela, Anu Joki, Terhi Koivumäki, Anu Katainen, Tea Lallukka

**Affiliations:** 1 Department of Public Health, Faculty of Medicine, University of Helsinki, Helsinki, Finland; 2 Faculty of Social Sciences, Tampere University, Tampere, Finland; 3 Faculty of Social Sciences, University of Helsinki, Helsinki, Finland

**Keywords:** health inequalities, obesity, qualitative research, socioeconomic disparities in health, systematic review

## Abstract

**Objective:**

The objective of this review is to synthesize the best available qualitative evidence on the weight management practices, views, and experiences of adults living with poor socioeconomic circumstances and obesity. Additionally, the review aims to deepen our understanding of the common narratives of obesity according to these people.

**Introduction:**

People living with poor socioeconomic circumstances are at increased risk of obesity, particularly in high-income countries, and their weight management practices (eg, weight-related behaviors) tend to be less healthy. Since prior research on socioeconomic inequalities in obesity is mostly from quantitative studies, the individual views and experiences related to weight management have been largely ignored. Thus, systematic qualitative evidence is needed on the weight management practices, views, and experiences of adults living with poor socioeconomic circumstances and obesity.

**Inclusion criteria:**

Qualitative studies examining adults (aged 18 to 74 years) living with poor socioeconomic circumstances and obesity, and conducted in high- and upper-middle-income countries will be considered. The phenomenon of interest is these people’s weight management practices, views, and experiences.

**Methods:**

Searches will be conducted in MEDLINE (Ovid), APA PsycINFO (Ovid), CINAHL (EBSCOhost), Scopus, Web of Science, and the Finnish health sciences database, MEDIC, restricted to the English and Finnish languages. Sources of unpublished studies and gray literature will include Google Scholar and ProQuest Dissertations and Theses. Two independent reviewers will screen the papers, assess methodological quality, and extract data following JBI’s procedures. The meta-aggregative approach will be used for data synthesis. Confidence in the findings will be assessed using the ConQual approach.

**Review registration:**

PROSPERO CRD42023407938

## Introduction

Socioeconomic inequalities in obesity are well established in high- and upper-middle-income countries: people with poor socioeconomic circumstances—such as those with low educational or income levels, low occupational position, or financial difficulties—are at higher risk of weight gain and subsequent obesity.^1,2^ However, in low- and lower-middle-income countries, there is an inverse relationship.^3^ Socioeconomic inequalities in obesity are observed in childhood,^4^ and these inequalities tend to persevere through late adulthood.^5,6^ This highlights the importance of considering life-course aspects to obesity and related inequalities. Given that obesity is a major risk factor for several chronic diseases, such as type 2 diabetes, cardiovascular diseases, and several common cancers,^7^ people with poor socioeconomic circumstances are also more vulnerable to obesity-related comorbidities. While the prevalence of obesity continues to increase worldwide, previous studies from high-income countries suggest that socioeconomic inequalities in obesity will also increase, or at least persist, in future years.^5,8^

Several explanations for the underlying mechanisms behind socioeconomic inequalities in obesity have been presented. For example, people with poor socioeconomic circumstances are more likely to face psychological and emotional distress, such as depression, chronic stress, low self-esteem and self-worth, and lack of support and cohesion, which may lead to maladaptive coping strategies.^9^ Unhealthy lifestyles, such as having dietary habits that are not consistent with recommendations and low physical activity, are more common among people with poor socioeconomic circumstances.^1,10^ This may be caused by multiple reasons, such as financial challenges to afford healthy foods, poor access to healthy foods and exercise facilities, challenges in adapting health knowledge (ie, health literacy), and challenges investing time in healthy lifestyles due to precarious work and stressful living conditions.^1,10^ Sociocultural factors also play a key role: for example, cultural norms for bodily appearance and advisable health-related behaviors are connected to social class.^11,12^ Quantitative studies suggest the causal pathway between socioeconomic circumstances and obesity are bidirectional; that is, poor socioeconomic circumstances predispose to obesity (ie, causation hypothesis)^13^ and obesity predisposes to poor socioeconomic circumstances (ie, selection hypothesis).^14^ Especially in women, who experience weight-related discrimination (eg, in the labor market) and bullying more often than men, the selection hypothesis is supported over the causation hypothesis.^14^

Although there is a large amount of research on socioeconomic inequalities in obesity, the current evidence is largely from quantitative studies.^1–4^ The suggested mechanisms and pathways between socioeconomic circumstances and obesity are mostly based on statistical models^9,10,13^; however, there are probably several unobserved factors that these models cannot capture. For example, individuals’ explanations for certain weight management practices (eg, weight-related behaviors, lifestyles, and weight loss strategies), individuals’ views (eg, perceptions and attitudes) and experiences toward weight management and obesity, as well as the heterogeneous weight histories and related narratives, are often neglected or cannot be addressed in quantitative studies. Weight-related stigma, which has been proposed to be a central driver of socioeconomic inequalities in obesity,^9,13^ could also be better understood via a more in-depth qualitative approach. Individual- and community-level interventions to reduce socioeconomic inequalities in obesity in the long term have not been as successful as societal-level interventions.^15^ However, qualitative studies that focus on individuals’ views and experiences on weight management and obesity might provide us with new perspectives on how individual- and community-level interventions would be more effective.

Quantitative studies have shown that weight management practices and views related to weight management are socioeconomically patterned.^16,17^ According to these studies, adults with poor socioeconomic circumstances—compared with those with more advantaged circumstances—are more likely to regard weight loss as expensive, laborious, and of low priority.^16^ Moreover, they are less likely to engage in weight management or join weight loss programs.^12,16,17^ They are also less likely to use weight loss strategies that are healthy and recommended (eg, exercising and reducing high-calorie foods) and are more likely to use unhealthy strategies (eg, pills, laxatives, fasting).^10,12,16,17^ A preliminary search of qualitative studies on adults living with poor socioeconomic circumstances and obesity revealed a number of factors influencing these people’s weight management practices, notably, problems with the accessibility to healthy foods, exercise facilities, and health care services^18–20^; financial barriers and food insecurity^18,20,21^; workload and responsibilities^20,21^; disordered eating behaviors^18^; and poor nutrition knowledge.^19^ Healthy lifestyles were seen as difficult, stressful, or not desirable enough to adhere to,^18–21^ and individuals were not supported by their social environment.^19–21^ Inconsistencies existed on the importance accorded to weight management and adherence to healthy lifestyles. So far, we lack systematic qualitative evidence to gather all these different aspects and findings on this topic.

A preliminary search of PROSPERO, *JBI Evidence Synthesis*, and MEDLINE (Ovid) was conducted and no current or in-progress systematic reviews on the topic were identified. The objective of this review is to synthesize the qualitative research evidence from high- and upper-middle-income countries on the weight management practices, views, and experiences of adults living with poor socioeconomic circumstances and obesity. Additionally, the review aims to deepen our understanding of the common narratives of obesity according to people with poor socioeconomic circumstances so that preventive acts can be developed to reduce obesity-related health inequalities and appropriate support for weight management.

## Review questions

- What are the weight management practices, views, and experiences of adults from high- and upper-middle-income countries who are living with poor socioeconomic circumstances and obesity?
- What are the common narratives of obesity according to these people?

## Inclusion criteria

### Participants

This review will consider studies that include adults (aged 18 to 74 years) living with poor socioeconomic circumstances and obesity from high- and upper-middle-income countries. We excluded older people (aged ≥75 years) since the relationship between body weight and health is affected by many diseases, disabilities, and health-related issues in this population group.^22^ Socioeconomic circumstances refer to the traditional measures of socioeconomic position, such as education, occupational class, and income, but also to the broader aspects of living conditions and material circumstances.^23^ Each socioeconomic indicator measures different aspects of socioeconomic circumstances, such as social status, prestige, material aspects, and literacy.^23^ This review will aim to capture all these different elements of socioeconomic circumstances. This will help us to gain a multifaceted comprehension of the complex phenomenon of socioeconomic inequalities in obesity. Thus, in this review, poor socioeconomic circumstances can consist of socioeconomic measures, such as having a low educational or income level, having a low occupational position, having financial difficulties or food insecurity, living in socioeconomically disadvantaged neighborhoods, living in poverty, or lacking material resources, but will not be limited only to these measures. Studies will be excluded if the contribution of the participant’s socioeconomic circumstances to the findings cannot be examined.

Obesity can be defined by body mass index (BMI; BMI ≥30.0 kg/m^2^ or equivalent, such as BMI ≥27.5 kg/m^2^ among Asian people), or by other anthropometric measures, such as waist circumference or waist-to-hip ratio.^24,25^ We will exclude studies where participants are not living with obesity (ie, participants living with underweight [BMI <18.5 kg/m^2^], normal weight [BMI 18.5–24.9 kg/m^2^], overweight without obesity [BMI 25.0–29.9 kg/m^2^]), or where the participants’ obesity status is not clearly stated or defined. However, if only a sub-sample of the study population is living with obesity and the findings can be evaluated separately, this sub-sample may be considered and analyzed. We will include all types of obesity; that is, class I, II, and III obesity (BMI 30.0–34.9 kg/m^2^, BMI 35.0–39.9 kg/m^2^, and BMI ≥40.0 kg/m^2^). It should be noted that people living with obesity also often live with comorbidities, such as type 2 diabetes, cardiovascular diseases, or musculoskeletal diseases.^7^ We will include studies in which participants are living with obesity and a specific comorbidity or disability, but the influence of these conditions to the findings will be considered in the analysis and discussion.

### Phenomena of interest

This review will consider studies that explore weight management practices (eg, weight-related behaviors and lifestyles, and weight loss and maintenance strategies) and views (eg, perceptions and attitudes) toward weight management. We will also consider studies that examine the experiences that influenced these peoples’ body weight and weight management, as well as their narratives of obesity.

### Context

This review will consider studies that are conducted in high- or upper-middle-income countries, as defined by the World Bank.^26^ The relationship between socioeconomic position and obesity is practically the opposite in high- and upper-middle-income countries compared with low- or lower-middle-income countries,^2,3^ which likely influences the manner in which socioeconomic differences in weight management practices, views, and experiences appear in these contexts. Thus, to avoid excessively heterogeneous findings due to contextual differences, we decided to exclude studies from low- and lower-middle-income countries.

### Types of studies

This review will consider primary studies that focus on qualitative data, including, but not limited to, designs such as phenomenology, ethnography, grounded theory, and narrative research. Mixed methods studies that include a qualitative research component will be also included. Quantitative studies, reviews of original studies, editorials, commentaries, conference abstracts, and letters will be excluded.

## Methods

The review will be conducted in accordance with the JBI methodology for systematic reviews of qualitative evidence.^27^ A protocol of the review was registered in PROSPERO (CRD42023407938).

### Search strategy

The search strategy will aim to locate both published and unpublished studies. An initial limited search of MEDLINE (Ovid) was undertaken to identify articles on the topic. The text words contained in the relevant articles and the index terms used to describe the articles were used to develop a full search strategy for MEDLINE (Ovid; see Appendix I). Then, the search strategy, including all identified keywords and index terms, was adapted for the databases to be searched in the review: APA PsycINFO (Ovid), CINAHL (EBSCOhost), Scopus, Web of Science, and the Finnish health sciences database, MEDIC. Additionally, sources of unpublished studies and gray literature will be searched via Google Scholar and ProQuest Dissertations and Theses. The first 30 pages will be screened in Google Scholar. The reference lists of all studies selected for critical appraisal will be screened for additional studies.

Studies published in English or Finnish will be included, since these are the languages spoken by the authors of this review. Any reports excluded on the basis of language will be reported in the final review. No time limit will be applied because the existing qualitative evidence on the topic is limited and we aim to include all available data on the topic. However, the interpretation and discussion of the results will consider possible temporal trends related to the phenomenon of interest.

### Study selection

Following the search, all identified citations will be collated and uploaded into the JBI System for the Unified Management, Assessment and Review of Information (JBI SUMARI; JBI, Adelaide, Australia)^28^ and duplicates removed. Following a pilot test, titles and abstracts will be screened by 2 independent reviewers (JS and AJ) for assessment against the inclusion criteria. Potentially relevant studies will be retrieved in full, and their citation details imported into JBI SUMARI. The full text of selected citations will be assessed in detail against the inclusion criteria by 2 independent reviewers (JS and AJ). Reasons for exclusion of full-text studies that do not meet the inclusion criteria will be recorded and reported in the systematic review. Any disagreements that arise between the 2 reviewers at any stage of the study selection process will be resolved through discussion or with a third reviewer (TK). The results of the search will be reported in full in the final systematic review and presented in a Preferred Reporting Items for Systematic Reviews and Meta-Analyses (PRISMA) flow diagram.^29^

### Assessment of methodological quality

Eligible studies will be critically appraised by 2 independent reviewers (JS and AJ) for methodological quality using the standard JBI critical appraisal checklist for qualitative research.^27^ Authors of papers will be contacted to request missing or additional data for clarification, where required. We will make up to 3 attempts to contact the authors within 3 weeks. Any disagreements that arise between the 2 reviewers will be resolved through discussion or with a third reviewer (TK). The results of the critical appraisal will be reported in narrative format and in a table. Following critical appraisal, studies that do not reach a quality threshold of 5/10—that is, studies that are low quality—will be excluded. This decision will be based on the assessment of the 2 independent reviewers.

### Data extraction

Data will be extracted by 2 independent reviewers (JS and AJ) using the standardized JBI data extraction tool.^27^ The extracted data will include specific details about the participants (eg, age, gender, ethnicity), context (eg, country, location, culture), study methods (eg, sampling and data analysis methods), and the phenomena of interest relevant to the review objective. Findings and their illustrations will be extracted verbatim and assigned a level of credibility. Any disagreements that arise between the 2 reviewers will be resolved through discussion or with a third reviewer (TK). Authors of papers will be contacted to request missing or additional data, where required. We will make up to 3 attempts to contact the authors within 3 weeks.

### Data synthesis

Qualitative research findings will, where possible, be pooled using JBI SUMARI with the meta-aggregation approach.^27^ This will involve the aggregation or synthesis of findings to generate a set of statements that represent that aggregation, through assembling the findings and categorizing these on the basis of similarity in meaning. These categories will then be subjected to a synthesis to produce a single comprehensive set of synthesized findings that can be used for evidence-based practice. Where textual pooling is not possible, the findings will be presented in narrative format. Only unequivocal and credible findings will be included in the synthesis.

### Assessing confidence in the findings

The final synthesized findings will be graded according to the ConQual approach for establishing confidence in the output of qualitative research synthesis and presented in a Summary of Findings.^30^ The Summary of Findings will include the key elements of the review and will detail how the ConQual score was developed. Included in the Summary of Findings will be the title, population, phenomena of interest, and context of the review. Each synthesized finding will then be presented, along with the type of research informing it, the score for dependability and credibility, and the overall ConQual score.

## Acknowledgments

Information specialist, Katri Larmo, from the Helsinki University Library for the helpful assistance with the search strategy.

## Funding

JS, AJ, and TL are supported by the Academy of Finland (Grant #1330527). AJ is also supported by the Juho Vainio Foundation (Grant #4709721). TK is supported by the Alli Paasikivi Foundation (Grant #230006).

## Author contributions

JS contributed to the conception and design of the study and the search strategy, and AJ, TK, AK, and TL critically reviewed them. JS performed the preliminary search and drafted the protocol; AJ, TK, AK, and TL critically reviewed it. All authors (JS, AJ, TK, AK, and TL) read and accepted the final version of the protocol.

## Appendix I: Search strategy

### MEDLINE (Ovid)

Search conducted on February 17, 2023

**Table:**

Search	Query	Records retrieved
#1	“socioeconomic factors”[MeSH] OR “social conditions”[MeSH] OR “social environment”[MeSH] OR “financial stress”[MeSH] OR “health inequities”[MeSH] OR “health equity”[MeSH] OR “social determinants of health”[MeSH] OR poverty[MeSH] OR “healthcare disparities”[MeSH] OR “working poor”[MeSH] OR “academic performance”[MeSH] OR socioeconomic* OR socio-economic* OR disadvantage* OR “social determinant*” OR “social position*” OR “social class*” OR “social status*” OR inequal* OR dispari* OR povert* OR “financial strain*” OR “financial stress*” OR “financial hardship*” OR “financial difficult*” OR “financial status*” OR “economic strain” OR “economic hardship*” OR “economic difficult*” OR “economic status*” OR “economic strain*” OR “income level*” OR “occupational position*” OR “occupational status*” OR “educational status*” OR “educational level*” OR “employment status*” OR “financial insecurit*” OR “economic insecurit*” OR inequit*	938,934
#2	overweight [MeSH] OR obes* OR overweight*	449,548
#3	“body weight changes”[MeSH] OR “weight perception”[MeSH] OR “weight manage*” OR “weight gain*” OR “weight loss*” OR “weight reduct*” OR “weight control*” OR “weight maintenance* OR “body weight change*” OR (weight* adj3 practice*) OR (weight* adj3 experienc*) OR (weight* adj3 view*) OR (weight* adj3 strateg*) OR (weight* adj3 habit*) OR (weight* adj3 behavio*) OR (weight* adj3 aspect*) OR (weight* adj3 explanat*) OR (weight* adj3 narrativ*) OR (weight* adj3 cause*) OR (weight* adj3 factor*) OR (weight* adj3 percept*) OR (weight* adj3 attitud*)	241,248
#4	“grounded theory”[MeSH] OR “qualitative research”[MeSH] OR “focus groups”[MeSH] OR “qualitative stud*” OR “qualitative method*” OR “qualitative research*” OR hermeneutic* OR ethnograph* OR ethnolog* OR phenomenolog* OR “discourse analysis” OR “narrative research*“ OR “narrative analysis” OR “content analysis” OR “thematic analysis” OR “action research*“ OR “participatory research*“ OR “mixed method*“ OR “focus group*” OR “semi-structured interview*“ OR “in-depth interview*“ OR “qualitative analys*“ OR “grounded theor*”	466,988
#5	#1 AND #2 AND #3 AND #4	940
Limited to full-text journal articles and to adult age group (18+ years), reported in English or Finnish.		137
